# The First 1000 Days: Maternal Nutrient Intake—A Window of Opportunity for Pulmonary Hypertension—A Narrative Review

**DOI:** 10.3390/nu18030424

**Published:** 2026-01-27

**Authors:** Alina-Costina Luca, Solange Tamara Roșu, Cosmin Diaconescu, Dana Elena Mîndru, Cristina Gavrilovici, Adriana Vizireanu, Viorel Țarcă, Eduard Vasile Roșu, Elena Țarcă

**Affiliations:** 1 Department of Pediatrics, Faculty of Medicine, Grigore T. Popa University of Medicine and Pharmacy, RO-700115 Iasi, Romania; 2 Department of Nursing, Faculty of Medicine, Grigore T. Popa University of Medicine and Pharmacy, RO-700115 Iasi, Romania; 3 Faculty of Medicine, Apollonia University, RO-700511 Iași, Romania; 4 Department of Surgery II—Pediatric Surgery and Orthopedics, Faculty of Medicine, Grigore T. Popa University of Medicine and Pharmacy, RO-700115 Iasi, Romania

**Keywords:** first 1000 days, pulmonary hypertension, maternal nutrition, polyphenols, thiamine

## Abstract

The first 1000 days of life, starting from conception to a child’s second birthday, constitute a pivotal period for fetal lung and pulmonary vascular development. Maternal nutrition during this period plays an important role in fetal growth, immune programming and organ development, including that of the pulmonary system. This narrative review consolidates evidence linking maternal nutrition and early-life nutrient intake during this period with the development of pulmonary hypertension in the newborn. We examine the influence of both nutrient deficiencies and excesses on fetal lung and vascular development. We performed a structured search of PubMed and Embase (conducted from February 2025 to March 2025) and screened reference lists. Twenty-eight peer-reviewed studies were included, comprising human clinical and observational evidence and studies on animal models. The findings suggest that imbalances in maternal diet can disrupt placental function, induce inflammation, and trigger epigenetic alterations, all contributing to pulmonary vascular dysfunction and increased pulmonary hypertension susceptibility in neonates. Notably, maternal undernutrition and thiamine deficiency during lactation have been directly linked to pulmonary hypertension in infants. Conversely, high-fat diets and excessive polyphenol intake have been associated with adverse fetal cardiovascular remodeling. While current evidence is primarily derived from animal models and observational studies, it highlights the urgent need for targeted nutritional strategies and clinical trials during pregnancy. Although human causality is unproven for most exposures, studying maternal nutrition in the first 1000 days could offer a cost-effective method for reducing the burden of pediatric pulmonary hypertension and its long-term consequences and for prospective trials aimed at preventing early-life pulmonary vascular disease.

## 1. Introduction

The First 1000 Days is a term that refers to the period between conception and two years after birth, culminating in a total duration close to 1000 days [1]. The relevance of the 1000-day period can be related to numerous factors, including growth and development, greater nutritional needs, higher vulnerability towards disease, heightened sensitivity to programming influences, coupled with total reliance on others for caregiving, nutrition, and social engagement [2,3]. Three key stages within the first 1000 days of human life present specific vulnerabilities: the embryonic stage, the perinatal period, and the lactation phase [4]. Every stage requires a vast amount of diverse nutrients, provided through placental transfusion, breastfeeding, and later through complementary foods [5].

Many studies highlight several mechanisms through which early-life nutrition determines future health outcomes [6,7,8]. These stem from the theory of nutritional programming introduced by the renowned physicist and epidemiologist David Barker, who described through his studies the association between an infant’s birth weight, largely a consequence of the mother’s dietary behavior, and diseases such as ischemic heart disease, type 2 diabetes, dyslipidemia, or hypertension [9].

In our review, we will assess the link between maternal nutrition in the first 1000 days and pulmonary hypertension.

To address the current fragmentation of evidence between nutrition in the first 1000 days, developmental lung biology and pulmonary vascular disease, we used pulmonary hypertension as the central outcome. Specifically, we focused on fetal hemodynamic alterations that can predispose to pulmonary hypertension, postnatal nutritional deficiencies that can present as acute infant pulmonary hypertension, and prenatal nutritional programming pathways that plausibly increase pulmonary vascular vulnerability. Our original contribution was an evidence synthesis that separates exposures with direct clinical links to neonatal PH from those supported mainly by mechanistic or indirect data, helping clinicians and researchers prioritize the most actionable hypotheses.

Pulmonary hypertension (PH) is a complex disease that occurs when blood pressure in the pulmonary arteries rises to abnormal levels, putting strain on the heart and lungs [10]. An important fact is that PH is being increasingly recognized in infants born prematurely. It is linked to a higher risk of mortality, bronchopulmonary dysplasia (BPD), and long-term heart and lung complications [11]. The overall rate of pulmonary hypertension of the newborn (PPHN) is 1.8 per 1000 live births. However, despite common assumptions, PPHN occurs more frequently in late preterm infants, with an incidence of 5.4 per 1000 live births. Among term infants, the incidence is 1.6 per 1000 live births. Reported mortality varies from 7.6% to 10.7%, depending on disease severity. Male newborns showed a greater likelihood than females, with an adjusted risk ratio of 0.8 (95% CI 0.7–0.8). African American infants had the greatest risk, followed by Hispanic and Asian newborns [12]. Persistent pulmonary hypertension of the newborn (PPHN) has a multifactorial etiology, and the mechanisms leading to persistently elevated pulmonary vascular resistance are complex and not fully understood. Clinically, distinct hemodynamic phenotypes can lead to acute pulmonary hypertension: (1) a predominantly pre-capillary phenotype with increased pulmonary vascular resistance (the most common); (2) a phenotype driven by increased pulmonary blood flow with pulmonary vascular remodeling, such as in left-to-right shunt lesions and arteriovenous malformations; (3) a post-capillary phenotype with increased pulmonary venous hypertension, for example, in obstructed total anomalous pulmonary venous connection or severe left ventricular dysfunction. Understanding the phenotype and underlying pathophysiology is important because these mechanisms imply different clinical trajectories and management priorities, and they also provide context for interpreting nutrition-related associations discussed in this review [13].

Many studies recognize dietary habits and nutritional status as key factors that can be modified to influence the emergence and evolution of respiratory illnesses such as pulmonary hypertension. They play a crucial role in regulating the immune system, which in turn affects respiratory health. Nutrients do more than just provide energy; they significantly contribute to overall lung function and health [14].

The primary goal of this study is to highlight the importance of nutrients and maternal dietary habits in the development of early-life pulmonary hypertension in offspring.

## 2. Methods

This narrative review was conducted using a structured search and screening approach to summarize evidence linking maternal and early-life nutrient exposure during the first 1000 days with pulmonary vascular outcomes in offspring, with an emphasis on neonatal pulmonary hypertension and persistent pulmonary hypertension of the newborn (PPHN).

### 2.1. Data Sources and Temporal Scope

Research was conducted by using PubMed and Embase from February 2025 to March 2025.

### 2.2. Search Strategy

Search concepts were combined using Boolean operators and included: pulmonary vascular outcomes (for example: pulmonary hypertension, pulmonary arterial hypertension, PH, PAH, lung health, lung disease, lung), timing and population (for example: pregnancy, pregnant women, fetal growth, pregnant, lactation, offspring, “first 1000 days”, mother) and nutrition-related exposures (for example: nutrition, diet, nutrient, vitamin, vitamin D, vitamin C, obesity, undernutrition, malnutrition and salt).

The search combined pulmonary vascular outcomes with pregnancy/early-life exposures and nutrition-related terms. An example PubMed strategy was: “(pulmonary hypertension) AND (first 1000 days) AND (nutrients)”. Equivalent terms and controlled vocabulary were used where available and adapted for each database. Reference lists of included full-text papers were hand-searched to identify additional relevant studies.

### 2.3. Eligibility Criteria

Eligible studies included peer-reviewed human studies, such as observational, interventional, case series reports, systematic reviews and meta-analyses relevant to fetal and neonatal pulmonary vascular development or neonatal pulmonary hypertension. Given the limited availability of direct human evidence for several exposures, animal studies (rodent and large-animal models) were also eligible when they addressed fetal pulmonary vascular development, ductus arteriosus constriction, pulmonary vascular remodeling, or experimental pulmonary hypertension relevant to early life.

We excluded non-English papers, editorials, commentaries, conference abstracts without accessible full text, and non-peer-reviewed sources. Studies focusing exclusively on pulmonary hypertension in pregnant women without fetal/neonatal pulmonary vascular outcomes were outside the scope of this review.

### 2.4. Study Selection and Synthesis

Titles/abstracts were screened for relevance, and potentially eligible studies underwent full-text review. The included evidence was narratively synthesized and organized into: exposures with direct clinical links to neonatal pulmonary hypertension, and exposures supported predominantly by preclinical, mechanistic, or indirect clinical data. Meta-analysis was not performed because of heterogeneity in exposures, populations, outcomes, and study designs.

Overall, 28 studies were included in the qualitative synthesis. Among these, 9 animal-model studies were included in the review due to the limited number of studies on human subjects.

## 3. Results

The targeted database search and reference-list screening identified key peer-reviewed papers relevant to maternal nutrition and pulmonary vascular and lung development. Twenty-eight studies were narratively synthesized as included evidence, comprising experimental studies (rodent/sheep/lamb models) on fetal lung development, ductus arteriosus constriction, vascular remodeling and pulmonary hypertension, and human clinical evidence linking nutrient deficiency to pulmonary hypertension in young infants (including breastfed infants), and pregnancy-related exposures to fetal or perinatal endpoints.

Because neonatal pulmonary hypertension encompasses distinct entities with different timing, the findings are presented by exposure window and endpoint: pregnancy-related exposures linked to fetal hemodynamic alterations and perinatal PPHN risk; lactation/early postnatal exposures linked to acute infant pulmonary hypertension presentations; and prematurity-associated pulmonary vascular disease evolving later in preterm infants (often in the context of BPD).

To strengthen conceptual clarity and maintain a consistent focus on early life pulmonary hypertension, the included evidence was grouped by the strength and directness of its link to pulmonary hypertension (see Table 1).

### 3.1. Nutrient Intake of Pregnant Women

The first period affecting lung health is between conception and birth. Dietary habits and pre-pregnancy metabolic conditions of pregnant women are crucial because of their ability to regulate gene expression in the placenta, influence organ formation, and impact metabolism and growth during critical stages. As a result, they can affect a child’s risk of developing chronic conditions like heart disease, diabetes, respiratory issues, immune disorders, and neuropsychiatric conditions. These risks exist regardless of whether the baby is born with a low birth weight or not [15].

A study conducted on a sample of 1003 pregnant women from the USA revealed multiple dietary patterns that could be associated with fetal development (see Table 2) [16].

In this cohort, inadequate intakes are revealed across multiple micronutrients, with nearly half of participants below recommended intake levels for magnesium (47.5%), vitamin D (46.4%), and vitamin E (43.3%), and more than one-third below recommended iron intake (36.2%). Additional shortfalls were observed for vitamin A, folate, calcium, vitamin C, vitamin B6, and zinc. This pattern suggests that micronutrient gaps are common even in a relatively young pregnant population and may co-occur rather than appearing as isolated deficiencies.

Concurrently, excess intake was frequent for some nutrients, most notably sodium (95%), and was also observed for vitamin K (47.9%), potassium (41.7%), folic acid (33.4%), and iron (27.9%). The co-existence of deficiencies and excesses within the same sample is consistent with heterogeneous dietary patterns and supplement use, reinforcing that “overall intake” in pregnancy may involve both underconsumption of nutrient-dense foods and overexposure to specific nutrients from fortified foods or supplements.

### 3.2. Essential Nutrients for Women During Pregnancy and Lactation

For the fetus to develop normally, the mother needs to consume a wide range of nutrients in adequate amounts throughout pregnancy (see Table 3). Even after the child is born, if the mother is breastfeeding, maternal diet strongly influences some milk components like milk fatty acids. In lactation, recommendations are explicitly provided for nutrients relevant to maternal stores and infant needs, including vitamin C, calcium, magnesium and zinc, and omega-3 (DHA) is highlighted as a priority during the infant’s first year [17].

Recent synthesis work on nutritional requirements in pregnancy and lactation emphasizes that nutritional planning should begin in the preconception period and that a balanced diet based on nutrient-dense foods is central to meeting increased maternal and fetal demands. Talebi et al. summarize that calorie intake needs to rise across pregnancy, with emphasis on adequate protein and healthy fats, and that lactation represents a distinct physiological state with continued nutritional demands to support milk production and maternal recovery [18].

**Table 3:** Estimated average nutrient requirements and recommendations for women during pregnancy and lactation (adapted from Jouanne et al.) [19].

Nutrient	Estimated Average Requirements (Pregnancy)	Lactation Recommendation
Vitamin A (μg/day)	550	10,000 IU/day or 25,000 IU/week or 200,000 IU once (deficiency only)
Vitamin D (μg/day)	10	10 µg/day (400 IU/day)
Vitamin E (mg/day)	12	-
Vitamin B1 (mg/day)	1.2	-
Vitamin B2 (mg/day)	1.2	-
Vitamin B3 (mg/day)	14	-
Vitamin B6 (mg/day)	1.6	-
Vitamin B9 (Folate, μg/day)	520	400 µg/day
Vitamin B12 (μg/day)	2.2	-
Vitamin C (mg/day)	70	130 mg/day
Calcium (mg/day)	800	1000 mg/day
Iodine (μg/day)	160	-
Iron (mg/day)	22	60 mg/day (for 3 months postpartum)
Magnesium (mg/day)	290	390 mg/day
Phosphorus (mg/day)	580	-
Selenium (μg/day)	49	-
Zinc (mg/day)	9.5	19 mg/day
Omega–3	100 mg/day of docosahexaenoic acid during infant’s first year	

### 3.3. Maternal High-Fat Diet

**Figure 1:**
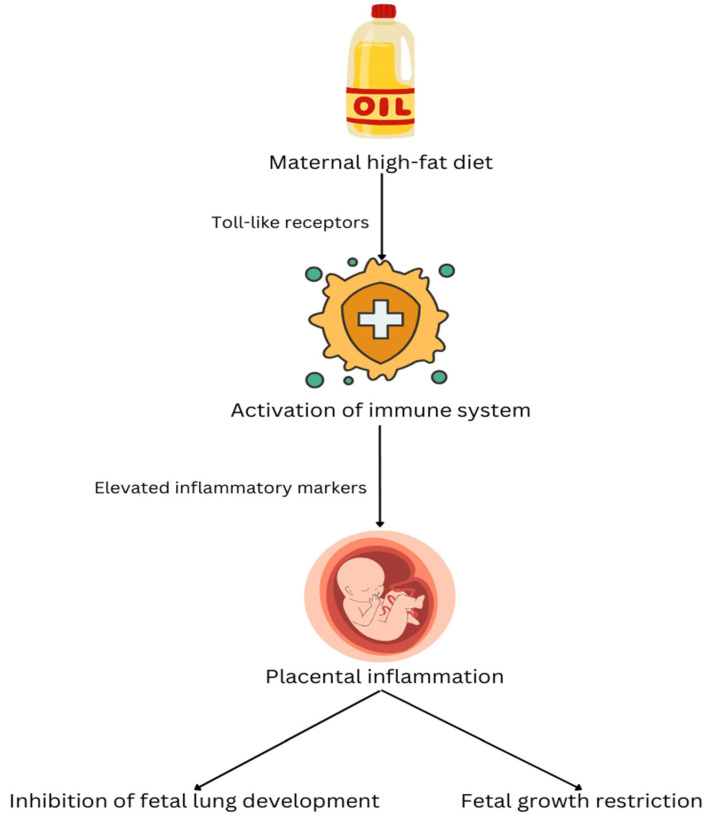
The mechanism by which a maternal high-fat diet affects the development of the fetal lungs.

To date, several studies stated that maternal obesity associated with a high-fat diet during pregnancy alters the fetal metabolic program through epigenetic modifications, which in turn raises the risk of obesity and metabolic diseases in the offspring by influencing energy regulation and lipid metabolism [20,21]. The Barker Hypothesis established that the intrauterine environment, particularly inadequate maternal nutrition, contributes to restricted fetal growth and multisystem developmental disabilities. Diets high in saturated fatty acids, for example, palmitic acid, have been implicated in promoting tissue inflammation through engagement of Toll-like receptors, leading to innate immune activation. Chronic consumption of a high-fat food has been shown to elevate circulating inflammatory markers in the maternal system, including serum amyloid A3, an acute-phase reactant protein. Importantly, fetal exposure to a maternal high-fat diet (independent of confounding factors such as maternal obesity and hyperinsulinemia) has been linked to placental inflammation. Given that placental function is essential for proper fetal development and that placental insufficiency represents the most prevalent cause of intrauterine growth restriction, these findings are particularly significant. In this context, the study conducted by Mayor et al. on mice provides promising evidence that maternal high-fat dietary intake induces inflammatory responses within the fetal-placental unit, ultimately contributing to fetal growth restriction and impaired fetal lung development (see Figure 1). Figure 1 summarizes an inflammatory pathway linking a maternal high-fat diet to adverse fetal outcomes in animal models. A high-fat diet may activate innate immune signaling (Toll-like receptor pathways), increase maternal inflammatory mediators, and promote placental inflammation. In turn, placental inflammation leads to fetal growth restriction and inhibition of fetal lung development, mechanisms that are biologically plausible upstream contributors to neonatal pulmonary vascular vulnerability [22].

Furthermore, maternal obesity is linked to a higher risk of congenital heart defects in infants (atrial septal defect, patent ductus arteriosus, aortic arch defects), possibly due to metabolic imbalances, placental dysfunction, and disrupted endothelial signaling during early fetal development. These factors may increase the likelihood of long-term cardiovascular issues in the child [23]. Another study conducted on mice revealed that maternal high-fat diet and perinatal inflammation contribute to pulmonary hypertension by triggering IL-6 trans-signaling, which leads to vascular remodeling and endothelial dysfunction [24].

On the positive side, vegetarian and vegan dietary patterns during pregnancy may be associated with lower maternal adiposity and cardiometabolic risk, which could indirectly influence inflammatory pathways relevant to fetal lung and vascular development. The Norwegian Nutrition Council has stated that vegetarian and vegan diets may be suitable in pregnancy and may reduce the risk of obesity and pre-eclampsia, while emphasizing monitoring of iodine, vitamin B12 and vitamin D to prevent clinically relevant deficiencies [25]. More recently, the ESPGHAN Nutrition Committee published a position paper based on a systematic search (10 included studies on approximately 1500 children) evaluating vegan diets in infants, children, and adolescents. The committee concluded that current evidence remains inconclusive regarding whether a strictly vegan diet consistently supports normal childhood growth (although available data did not show significant differences in height or BMI z-scores versus omnivorous peers). ESPGHAN recommends regular monitoring of dietary intake, growth, and nutritional status in children on vegan diets, with structured dietetic counseling and supplementation of specific micronutrients (particularly vitamin B12) alongside attention to nutrients commonly at risk (protein quality, omega-3, calcium, and iron) [26].

### 3.4. Maternal Undernutrition

If the effects of obesity and a high-fat diet during pregnancy are important, we must also not ignore the effects of undernutrition. Rexhaj et al. demonstrated in their study on the mouse model that maternal undernutrition in pregnancy resulted in pulmonary endothelial dysfunction in vitro, expressed as increased hypoxic pulmonary hypertension (35.8 ± 3.5 vs. 30.8 ± 4.9 mmHg, *p* = 0.002, restrictive diet vs. controls) and right ventricular hypertrophy (RV-to-LV + S ratio: 0.329 ± 0.028 vs. 0.289 ± 0.049, *p* = 0.013, restrictive diet vs. controls) in vivo. This pulmonary vascular dysfunction was linked to changes in lung DNA methylation [27]. Additionally, a study by Barker et al. emphasized that undernutrition during pregnancy can lead to reduced fetal growth and placental dysfunction, both facts being linked to pulmonary hypertension [28,29].

### 3.5. Polyphenol-Based Diet During Pregnancy

**Figure 2:**
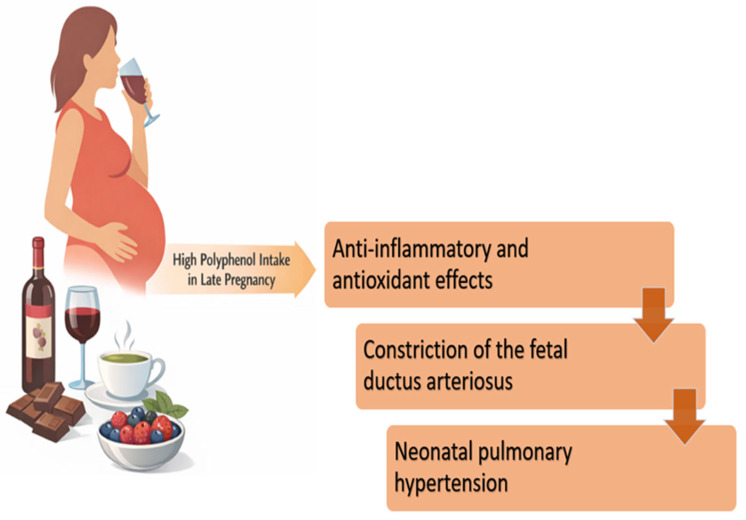
Examples of polyphenol-containing foods and beverages commonly consumed emphasize that polyphenol exposure in pregnancy arises from everyday dietary choices. The figure is meant to be illustrative (not exhaustive) and supports dietary history and counseling. It is not a recommendation for the avoidance of the foods shown. In the context of the experimental findings described above, it illustrates the concept that excessive polyphenol intake in late pregnancy could be relevant when fetal ductal constriction is suspected. This figure illustrates common polyphenol-rich foods for awareness in the context of ductal constriction risk, not for avoidance unless clinically indicated. (adapted from Jawhara S.) [31].

Another relevant aspect worth discussing is the anti-inflammatory potential of a polyphenol-rich diet. Various foods and beverages (including herbal teas, grapes and their derivatives, oranges, chocolate, and a wide range of fruits) contain high levels of polyphenols and are commonly consumed during pregnancy (see Figure 2, which presents the possible adverse effects of polyphenol-containing foods that are commonly included in pregnant women’s routine diet). While polyphenols are generally associated with positive health benefits, as outlined earlier, emerging evidence suggests that their consumption in the later stages of pregnancy (particularly during the third trimester) may pose risks to fetal health. This concern arises from the anti-inflammatory and antioxidant effects of polyphenols on the fetal ductus arteriosus. Constriction of the fetal ductus arteriosus is a recognized risk factor for the development of neonatal pulmonary hypertension, a condition associated with significant clinical complications. To explore this possibility, a series of experimental studies was conducted on fetal lambs and observed that maternal intake of polyphenol-rich substances was associated with echocardiographic signs of ductal constriction in the fetus [30].

### 3.6. High Salt Diet During Pregnancy and Lactation

When discussing condiments consumed during pregnancy, it is not possible to exclude salt, which is likely present in most households. Piecha et al. discovered that when rats are exposed to a high-salt diet during pregnancy and lactation, their offspring (after 12 weeks of age) exhibit vascular changes characterized by increased wall thickness without changes in luminal area in the central and muscular arteries of both the systemic and pulmonary circulation [32]. Another study conducted on rats revealed that consuming too much salt during pregnancy led to left and right ventricular hypertrophy in male newborns. Also, in the same study, the influence of salt consumption on the renal protein expression of angiotensin II receptors was observed. This may explain the cardiomyocyte hypertrophy in both ventricles [33].

### 3.7. Thiamine Deficiency During Breastfeeding

Another important aspect this review highlights is nutritional deficiency during breastfeeding. Currently, there are multiple studies on the impact of deficiencies in certain nutrients, such as vitamin D or vitamin C, but the less common deficiencies, such as thiamine deficiency, should not be overlooked either. A lack of thiamine is known to lead to severe pulmonary hypertension and acute right-sided heart failure. Sastry et al. conducted a study on 250 infants (mean age 3.2 ± 1.2 months) with severe pulmonary hypertension that revealed a thiamine deficiency. It was found that women from developing countries who were breastfeeding typically consumed polished rice, spicy soup, and low amounts of vegetables, fruits, cereals, and milk. This led to thiamine deficiency in infants who were dependent on their mothers’ nutrient intake. Pulmonary hypertension fully resolved in 92% (231 out of 250) of cases following thiamine supplementation [34]. Another study also highlighted the fact that thiamine deficiency is often a missed diagnosis due to the absence of classic symptoms. Thiamine deficiency that leads to cardiovascular dysfunction is known as beriberi. Most patients with beriberi exhibit rapid breathing, chest indrawing, tachycardia, and cardiomegaly with dilatation of the right heart chambers and pulmonary hypertension [35]. Both studies concluded that breastfed infants with thiamine deficiency should receive thiamine supplementation to reverse the effects of pulmonary hypertension and congestive cardiac failure [34,35]. The thiamine-responsive acute pulmonary hypertension of infants should be considered as a possible diagnosis in at-risk infants presenting with respiratory failure when all of the following criteria are met: (1) a recent diagnosis of pulmonary hypertension with no prior history of congenital heart defects or chronic lung conditions, (2) metabolic acidosis accompanied by elevated lactate levels, (3) absence of any other identifiable cause such as sepsis, (4) maternal diet history indicating potential thiamine deficiency in an infant who is exclusively or primarily breastfed, and (5) a prompt improvement following intravenous thiamine administration [36].

### 3.8. Vitamin D

The link between vitamin D and pulmonary dysfunction is well studied. The earliest studies on vitamin D supplementation during pregnancy were conducted in the early 1980s, primarily in Europe [37]. Vitamin D plays a crucial role in early fetal lung development, and this impact cannot be compensated for by beginning supplementation late in the first trimester [38,39]. Additionally, a healthy maternal immune response to the placenta is associated with vitamin D, as it plays a key role in modulating immune function [40]. During pregnancy, the maternal requirements of vitamin D are increased because of several factors in the vitamin D metabolism [41]. An interesting study by Cookson et al. reported that pulmonary hypertension can be prevented with intra-amniotic vitamin D, which helps preserve lung development in experimental bronchopulmonary dysplasia caused by intra-amniotic sFlt-1 [42].

### 3.9. Vitamin C

As specified in Table 2, vitamin C is an essential nutrient during pregnancy and breastfeeding. In studies on sheep and rats, maternal administration of Vitamin C protected against fetal growth restriction and the development of cardiovascular dysfunction in adult offspring [43]. The beneficial effects of vitamin C appear to be especially significant when consumed by pregnant women who smoke. Nicotine is a significant controller of fetal lung development. The fetuses of smokers are exposed to nicotine throughout pregnancy, and various studies using animal models, including primates, unequivocally state that nicotine slows fetal lung development [44]. A meta-analysis of the effects of vitamin C supplementation for pregnant smokers concluded that the offspring supplemented with vitamin C had improved Forced Expiratory Flow between 25 and 75% and Forced Vital Capacity compared to those not supplemented with vitamin C [45]. Evidence from multiple studies of vitamin C supplementation (500 mg/day) to pregnant smokers unable to quit suggests that vitamin C can improve lung health by preventing some of the effects of in utero smoke exposure [46].

## 4. Discussion

We synthesized evidence on maternal and early-life nutrition in the first 1000 days through the specific lens of offspring pulmonary hypertension, including primarily those pathways that can plausibly influence pulmonary vascular development and postnatal pulmonary vascular adaptation. To address the conceptual depth of literature about “The First 1000 Days”, we explicitly tiered the evidence by the directness of its association with neonatal PH/PPHN (see Table 1).

For many common nutritional exposures, the evidence base is dominated by animal models and indirect human data. These studies converge on a biologically coherent pathway in which altered maternal metabolic status promotes systemic and placental inflammation, disrupts placental perfusion and nutrient transport, and induces epigenetic changes that can impair fetal lung development and pulmonary endothelial function. Excessive exposure to polyphenol-rich foods and beverages late in pregnancy has been associated with fetal ductus arteriosus constriction in experimental and clinical fetal echocardiography studies, a recognized upstream mechanism that can precipitate PPHN. Thiamine deficiency in exclusively breastfed infants has been repeatedly reported to present as acute severe PH with right heart failure and metabolic acidosis, and to be rapidly reversible with thiamine supplementation. High-salt exposure and vitamins D and C are presented as plausible modulators with limited PH-specific human outcome data.

### 4.1. Exposures with Direct Clinical Relevance to Neonatal PH

Across the included literature, two nutritional exposures stand out as having the clearest and most actionable links to neonatal pulmonary hypertension. First, thiamine deficiency in exclusively breastfed infants has been repeatedly reported to present as acute severe PH with right heart failure and metabolic acidosis, and to be rapidly reversible with thiamine supplementation. This finding underscores the clinical importance of reviewing maternal dietary history and considering empiric thiamine in appropriate high-risk presentations, while at the same time pursuing standard evaluations for other causes of neonatal PH. Practical screening questions in suspected thiamine-responsive infant PH should include maternal dietary patterns, prolonged postpartum dietary restriction, hyperemesis or low intake during pregnancy/lactation, and exclusive or predominant breastfeeding without maternal supplementation. Clinically, thiamine deficiency may be considered in infants with acute PH accompanied by right-sided failure signs (tachycardia, hepatomegaly, cardiomegaly/RV dilation), and no alternative explanation after initial evaluation, given the low risk and potential reversibility reported in the literature.”

Second, excessive exposure to polyphenol-rich foods and beverages late in pregnancy has been associated with fetal ductus arteriosus constriction in experimental and clinical fetal echocardiography studies, a recognized upstream mechanism that can precipitate PPHN. The key implication is not a blanket avoidance of polyphenol-containing foods, but targeted dietary assessment and counseling when fetal ductal constriction is suspected or detected.

### 4.2. Prenatal Nutritional Programming: Placental Inflammation, Growth Restriction, and Pulmonary Vascular Vulnerability

For many common nutritional exposures (maternal obesity/high-fat diet and maternal undernutrition), the evidence base is dominated by animal models and indirect human data. These studies converge on a biologically coherent pathway in which altered maternal metabolic status promotes systemic and placental inflammation, disrupts placental perfusion and nutrient transport, and induces epigenetic changes that can impair fetal lung development and pulmonary endothelial function. Although direct human causal data linking these exposures to neonatal PH remain limited, this programming framework is consistent with clinical observations that placental dysfunction and fetal growth restriction are associated with bronchopulmonary dysplasia and later pulmonary vascular disease in preterm infants.

### 4.3. Salt and Micronutrients: Plausible Modulators, Limited PH-Specific Human Outcome Data

High-salt exposure during pregnancy and lactation has been associated with vascular structural changes in offspring in animal studies, including in the pulmonary circulation, suggesting a potential role for early-life dietary sodium as a modifier of vascular development. Similarly, vitamins D and C have well-described roles in immune modulation and lung maturation, and experimental work supports a potential impact on pulmonary vascular development in specific contexts. However, in most instances, the available human evidence relates to respiratory or developmental endpoints rather than PH as a primary outcome [43]. These data should therefore be interpreted as supporting biological plausibility rather than establishing human causality for neonatal PH.

### 4.4. Clinical and Research Implications

From a clinical standpoint, our synthesis emphasizes two high-yield opportunities: (1) incorporating maternal dietary history (including culturally patterned diets and potential micronutrient gaps) into the evaluation of infants with otherwise unexplained acute PH and metabolic acidosis, and (2) considering dietary polyphenol exposure during the third trimester as a modifiable factor in suspected fetal ductal constriction. As a broader dietary-pattern consideration relevant to micronutrient adequacy in early life, recent ESPGHAN guidance highlights that vegan diets in pediatric age require structured professional support, because the available evidence base is limited and largely observational, with inconclusive data on whether strictly vegan diets consistently support optimal growth across childhood. From a research perspective, prospective pregnancy and birth cohorts with standardized nutritional exposure measurement and harmonized PH-related endpoints (PPHN, echocardiographic pulmonary pressure estimates, and preterm BPD-PH phenotypes) are needed to move beyond mechanistic plausibility.

Interpretation of observational associations between maternal diet and offspring pulmonary vascular outcomes is limited by confounding and measurement bias. Maternal comorbidities (diabetes, obesity, infections, hypertensive disorders), socioeconomic status and food access, smoking, and concurrent supplementation or multiple micronutrient deficiencies may co-vary with dietary patterns and independently influence fetal growth, placental function, and neonatal respiratory adaptation. Additionally, dietary exposure assessment often relies on recall-based instruments prone to misclassification. Future cohorts should incorporate standardized dietary instruments complemented by biomarkers, prospectively capture key comorbidities and social determinants, and pre-specify PH-relevant neonatal endpoints to reduce residual confounding.

## 5. Limitations of the Study

This paper is a narrative review and therefore does not include a formal risk-of-bias assessment or a quantitative meta-analysis. Importantly, much of the available evidence is not directly linked to clinically adjudicated pulmonary hypertension in infants; several exposure domains are supported predominantly by animal studies or retrospective human analyses, and large-scale longitudinal human research with PH/PPHN as a primary endpoint remains limited. The included studies are heterogeneous with respect to exposure definitions (dietary patterns vs. biomarkers vs. supplementation), outcome definitions (fetal ductal constriction, PPHN, experimental pulmonary vascular remodeling), and populations (term vs. preterm; animal models vs. human cohorts), which constrains cross-study comparability. Observational human data are also vulnerable to residual confounding and measurement bias, while publication bias may preferentially amplify positive mechanistic findings in small studies. In addition, translational uncertainty from animal models to human pregnancy is substantial, given species differences in placentation, timing of lung development, and exposure dosing that may not reflect real-world intake. Finally, several clinically salient signals (thiamine-responsive PH) rely partly on case series or single-center cohorts, which can overestimate effect sizes and limit generalizability. Accordingly, we emphasized transparent evidence tiering (Table 3) and consistently used cautious, non-causal language where human causal evidence is lacking, underscoring the need for prospective multicenter studies with standardized exposure assessment and PH-relevant outcome definitions.

## 6. Prospects for Future Research

Future work should prioritize (1) prospective studies that pair standardized nutritional exposure assessment (dietary instruments, biomarkers, and supplement use) with clearly defined PH-related endpoints in the newborn and preterm infant; (2) mechanistic and translational studies integrating placental pathology, inflammation markers, and epigenetic profiling with fetal and neonatal pulmonary vascular phenotyping; (3) pragmatic supplementation or dietary-intervention trials in well-defined high-risk settings, such as populations with endemic thiamine deficiency or fetuses with suspected ductal constriction.

## 7. Conclusions

Maternal and early-life nutrition during the first 1000 days is a potentially modifiable set of exposures with relevance to fetal lung development and pulmonary vascular adaptation. In the current literature, the most clinically actionable evidence relates to thiamine deficiency in breastfed infants presenting with acute severe pulmonary hypertension that is often rapidly thiamine-responsive and late-pregnancy polyphenol-associated fetal ductal constriction as a mechanistic precursor to PPHN. In contrast, links between obesity/high-fat dietary exposure, undernutrition, high salt intake, and vitamins D and C with offspring pulmonary hypertension are supported mainly by preclinical, mechanistic, or indirect clinical data and should be interpreted as biological plausibility rather than proven human causality. Well-designed prospective cohorts and targeted intervention studies are required to define causal effects and preventive strategies.

## Figures and Tables

**Table 1:** Evidence map of nutrition-related exposures and pulmonary hypertension-relevant outcomes in offspring.

Exposure	Main Evidence Base	Pulmonary Hypertension-Relevant Outcome
Thiamine deficiency during exclusive breastfeeding	Human prospective/observational evidence + case series; rapid response to supplementation	Acute severe infant PH with right heart failure; high reversibility with thiamine
Polyphenol-rich intake in late pregnancy (3rd trimester) leading to ductal constriction	Human fetal echo studies + clinical reports; experimental/large-animal support	Fetal ductal constriction and downstream neonatal PH/PPHN risk
Maternal high-fat diet/obesity (during pregnancy)	Studies on animal models; indirect human associations via inflammation and placental dysfunction	Impaired fetal lung development, pulmonary vascular remodeling; biological plausibility for neonatal pulmonary vascular vulnerability
Maternal undernutrition/fetal growth restriction	Animal models and literature about placental dysfunction related to PH	Endothelial dysfunction and increased hypoxic PH in offspring models; plausibility for BPD-PH risk pathways
High salt intake during pregnancy and lactation	Animal models	Pulmonary and systemic vascular structural changes in offspring; plausibility for altered pulmonary vascular reactivity
Vitamins D and C (deficiency or supplementation)	Mixed: mechanistic and observational; targeted experimental models	Potential effects on lung maturation, immune modulation, and pulmonary vascular development; limited direct human PH outcomes

**Table 2:** Nutrient intake deficiencies and excesses in 1003 pregnant women with a mean age of 28 years from the USA (data adapted from Bailey R. et al. [16]).

Nutrient	Deficiency/Excess	Percentage	Standard Error
Magnesium	Deficiency	47.5	2.8
Vitamin D	Deficiency	46.4	2.7
Vitamin E	Deficiency	43.3	2.7
Iron	Deficiency	36.2	2.8
Vitamin A	Deficiency	15.5	2.1
Folate	Deficiency	16.4	1.6
Calcium	Deficiency	12.9	2.4
Vitamin C	Deficiency	11.5	1.9
Vitamin B6	Deficiency	11.5	1.5
Zinc	Deficiency	10.9	1.9
Potassium	Excess	41.7	2.9
Choline	Excess	7.9	3.2
Vitamin K	Excess	47.9	4.3
Sodium	Excess	95	2.2
Folic Acid	Excess	33.4	2.8
Iron	Excess	27.9	2.8
Calcium	Excess	3	0.8
Zinc	Excess	7.1	1.6

## Data Availability

No new data were created or analyzed in this study. Data sharing is not applicable to this article.
